# The association between physical activity and cardiovascular disease under indoor air pollution among middle-aged-to-elderly people: a sex-specific analysis from CHARLS

**DOI:** 10.1186/s12889-026-27175-w

**Published:** 2026-03-30

**Authors:** Huilong Xie, Bo Chen, Fan Xia, Jingbo Xia

**Affiliations:** 1 Guangdong Provincial Key Laboratory of Physical Activity and Health Promotion, Guangzhou Sport University, No.1268, Guangzhou Avenue Middle, Tianhe District, Guangzhou, Guangdong 510500 China; 2 Department of Exercise Physiology, Beijing Sport University, Beijing, China; 3 Graduate Department, Harbin Sport University, Harbin, Heilongjiang China

**Keywords:** Physical activity, indoor air pollution, cardiovascular diseases, middle-aged and elderly people

## Abstract

**Background:**

The associations between physical activity (PA) and outdoor air pollution with cardiovascular disease (CVD) are well established, but evidence regarding indoor air pollution remains scarce. This study examined sex-specific associations between PA and CVD in the context of indoor air pollution resulting from solid fuel use.

**Methods:**

This nationwide cross-sectional study analyzed data from 18,640 middle-aged and older adults from the 2018 wave of the China Health and Retirement Longitudinal Study. PA was measured using the International Physical Activity Questionnaire. Information on solid fuel use was obtained through self-reported primary sources of cooking and heating fuels. CVD was defined based on self-reported physician diagnoses of heart diseases or stroke.

**Results:**

Participants with higher PA levels exhibited a lower CVD prevalence, whereas all types of solid fuel use were associated with higher prevalence, with the highest prevalence observed among mixed solid fuel users. Physically inactive individuals generally exhibited the highest prevalence across all fuel-use groups. Exposure-response analyses revealed that PA was inversely associated with CVD prevalence among male users of clean energy, coal, and crop residue/wood burning, and among female users of clean energy and crop residue/wood burning (all P for overall < 0.05). Among males, PA significantly mediated the relationship between solid fuel use and CVD, with opposing statistical mediation directions observed for coal (5.07%) and crop/wood (–18.99%). In females, significant statistical mediation was observed only for crop/wood use (-16.89%).

**Conclusions:**

This cross-sectional study suggests that the inverse association between PA and CVD prevalence may vary by sex and solid fuel type. PA was associated with lower CVD prevalence across all fuel types in males, but only among females using clean energy and crop residue/wood burning. These findings highlight the need for targeted health policies for older adults undergoing energy transition.

**Supplementary Information:**

The online version contains supplementary material available at 10.1186/s12889-026-27175-w.

## Introduction

 While outdoor air pollution has been widely studied, research indicates that people spend over 90% of their time indoors, and vulnerable populations, particularly the elderly, spend more time indoors and higher levels of household air pollution exposure [1–3]. An estimated one-third of the world’s population still relies on traditional solid fuels—including charcoal, firewood, crop residues, and animal dung—for household cooking [4]. Solid fuel combustion is the major source of indoor pollution, which releases toxins associated with adverse cardiovascular effects [5, 6]. Cardiovascular disease (CVD), a leading global public health threat, has been shown to be influenced by exposure to such sources [6]. Despite increased policy support from local governments to promote the use of clean energy [7], lower-income households are still more likely to rely on polluting fuels (e.g., coal and biomass fuels) and reside in poorly ventilated environments, thereby exacerbating health inequalities [8, 9].

Physical activity (PA) has been demonstrated to confer cardiovascular protection through multiple mechanisms [10–13]. In contrast, solid fuel use for cooking or heating is associated with decreased health-related quality of life and even an increased risk of CVD. Solid fuels, mainly coal and crop residue/wood burning [14, 15], release different pollutants that may trigger distinct cardiovascular responses. It remains unclear whether PA, in the context of air pollution exposure, retains its protective effects [16], becomes less effective [17], or even increases cardiovascular risk [18], as some studies have suggested in the context of outdoor air pollution. Notably, sociobehavioral and physiological factors—such as the division of household tasks and hormonal differences—may contribute to gender differences in aging [19], CVD progression [20], and the cardiovascular response to PA [21, 22], potentially leading to unequal household pollution exposure [23].

Evidence regarding the association between PA and CVD in the context of indoor air pollution remains scarce. To address these knowledge gaps, we conducted a sex-stratified analysis using a nationally representative sample of middle-aged and older adults in China to investigate the associations between PA, different solid fuel sources, and CVD prevalence. We assessed the independent and joint association of PA with CVD prevalence in the context of indoor air pollution, characterized their exposure–response relationships, and examined whether PA statistically mediated the association between indoor air pollution and CVD prevalence.

## Method

### Study population

The China Health and Retirement Longitudinal Study (CHARLS) is a nationally representative longitudinal survey targeting individuals aged 45 years and older in China (https://charls.pku.edu.cn/). It employed a multistage, stratified sampling strategy with probability proportional to size, encompassing 150 counties or districts and 450 urban and rural communities across 28 provinces. Detailed methodology had been documented elsewhere [24]. The study protocol was approved by the Institutional Review Board of Peking University (IRB00001052-11015) and adhered to the ethical standards outlined in the Declaration of Helsinki.

For the present analysis, we utilized data from the 2018 CHARLS wave, which collected extensive information on participants’ PA. Individuals were excluded if they were under 45 years old or lacked complete data on CVD, PA, or essential covariates. After applying these exclusion criteria, a final sample of 18,640 participants was included in the analysis. A detailed overview of the participant selection process is provided in Supplementary Fig. 1.

### Self-reported physical activity

PA was assessed using a structured questionnaire derived from the International Physical Activity Questionnaire (IPAQ) [25, 26]. Participants provided self-reported data regarding how many days per week they engaged in PA, the duration per time, and the intensity level, which was categorized as light, moderate, or vigorous. To estimate total weekly duration, reported daily activity times were classified into five intervals: less than 10 min, 10–29 min, 30–119 min, 120–239 min, and 240 min or more. Following established methodologies [27], a representative median value was assigned to each time category, with 0 min and 240 min assigned to the lowest and highest groups, respectively. Weekly duration was subsequently computed by multiplying the frequency of activity by the median duration of the respective time group. Given that light-intensity activities may not offer comparable health advantages to moderate-to-vigorous physical activity (MVPA) [28, 29], only time spent in moderate and vigorous activities was summed to reflect total effective activity duration.

The intensity of PA was further quantified using metabolic equivalent tasks (METs) values [30]: 8.0 METs for vigorous activity and 4.0 METs for moderate activity, as previous literature [31]. Total PA volume, expressed in METs per week, was calculated using the formula: (8.0 × time of vigorous PA) + (4.0 × time of moderate PA). According to the WHO Guidelines on PA and Sedentary Behaviour [32] and IPAQ classification standards [25], higher levels of PA—specifically more than 300 min of moderate-intensity activity, more than 150 min of vigorous-intensity activity, or an equivalent combination per week—are recommended for additional health benefits. First, participants who reported no MVPA were classified as inactive. Second, to differentiate the active group, a threshold of 1200 MET-minutes per week (equivalent to 300 min of moderate or 150 min of vigorous PA) was applied; those exceeding this threshold were classified as physically active, with all remaining participants considered insufficiently active.

### Indoor air pollution

Indoor air pollution exposure was indirectly estimated based on the type of household fuels used for cooking and heating. Participants in the CHARLS survey were asked to report their primary sources of fuel for both cooking and heating purposes. Coal, crop residue/wood burning were considered “solid fuel”, and clean fuel was defined as the use of solar, natural gas, liquefied petroleum gas, or electricity for heating or cooking [33]. Mixed solid fuel usage was defined as using two solid fuel types for either heating or cooking.

### Cardiovascular diseases

The primary outcome of this study was the prevalence of CVD, defined as the presence of heart diseases (including angina, heart attack, congestive heart failure, and other heart problems) or stroke. In accordance with established precedents [34], participants were inquired whether they were diagnosed with heart disease or stroke by physician.

### Assessment of covariates

Demographic characteristics included age (years), sex (female, male), area of residence (rural, urban), and marital status (married or partner, others). Socioeconomic status was represented by education level (primary school and below, middle school, high school and above) and household expenditure per capita (low, medium, high), which can better symbolize the real economic status in China [35]. Health and behavioral factors included smoke (no, yes), drink (no, yes), and sleep duration (< 7 h, ≥ 7 h). Environmental covariates included installation of air purifier (no, yes) and air quality index (AQI) (units). AQI was retrieved from the Science Data Bank (https://www.scidb.cn/en). The AQI is an integrated measure representing the ambient air pollution level, derived from the concentrations of six major pollutants—PM_2.5_, PM_10_, CO, SO_2_, NO_2_, and O_3_ —based on threshold criteria established by the GB3095-2012 national air quality standards [36].

### Statistical analyses

Given the biological differences between males and females in aging trajectories [19], CVD progression [20], and the cardiovascular response to PA [21], all statistical analyses were stratified by sex. Descriptive statistics were reported separately for females and males, using means with standard deviations, counts with percentages, or medians with interquartile ranges (25th and 75th percentiles), as appropriate. To examine the relationship between PA and CVD prevalence, as well as the association between exposure to indoor pollution and CVD prevalence, multivariable logistic regression analyses were conducted. The initial model adjusted for demographic and socioeconomic factors, including age, sex, residential area, marital status, and household expenditure per capita. In the second model, lifestyle related variables include smoking status, alcohol consumption, and sleep duration. The fully adjusted model further incorporated environmental factors, namely air purifier usage and AQI. Odds ratios (ORs) and 95% confidence intervals (CIs) were calculated, along with P values for trend. To assess potential multicollinearity among covariates, a correlation matrix was generated, revealing no substantial collinearity between variables.

Joint analyses were performed by categorizing both indoor pollution exposure and MVPA into three groups: inactive, insufficiently active, and physically active. Participants who were physically active and used clean household energy served as the reference group. Furthermore, to explore potential dose–response relationships, restricted cubic spline was applied within fully adjusted logistic regression models to assess the linear associations between MVPA duration and the prevalence of CVD, stratified by levels of household fuel use.

To investigate whether MVPA serves as a mediator in the relationship between indoor air pollution and CVD prevalence, we conducted a mediation analysis. The fully adjusted logistic regression model included both the primary exposure variable—solid fuel use—and the mediator, characterized by MVPA duration, to estimate the total and indirect effects. The mediation effect was quantified as the proportion of the indirect pathway relative to the total effect, expressed as a mediation percentage. The statistical significance of this mediating pathway was evaluated via bootstrap resampling (times = 1000) [37]. In addition, generalized additive models were employed to explore potential nonlinear relationships between household fuel type and MVPA levels. All statistical analyses were conducted using R software (version 4.3.3). Two-sided tests were applied throughout, with statistical significance defined as a P value < 0.05.

### Sensitive analyses

We performed a series of sensitivity and robustness analyses to enhance reliability. First, due to the limited categories provided by the WHO guidelines, we employed an alternative PA classification: inactive (no MVPA), insufficiently active (any MVPA below the median), and physically active (any MVPA at or above the median) (Supplementary Tables 6–7). Second, we employed structural equation modeling as an alternative mediation method for robustness checks (Supplementary Table 8). Third, we further adjusted for indoor ventilation (good: wind pressure-dominant vs. poor: thermal pressure-dominant) based on building type (Supplementary Tables 9–11) [38, 39]. Finally, to address potential bias introduced by non-response and individual-level censoring, we incorporated sampling weights into the analysis (Supplementary Tables 12–14). Sensitivity analyses produced similar findings, collectively supporting the robustness of the main results.

## Results

### Characteristics of the study participants

Table 1 presented the characteristics of the 18,640 participants included in the study. The mean age was 61.66 years. Of the total sample, 9,735 participants (52.2%) were female and 8,905 (47.8%) were male. A total of 4,572 participants (24.5%) had a diagnosis of CVD. Regarding fuel usage, most participants reported using clean energy (12,153 participants, 65.2%), followed by wood (4,715 participants, 25.3%), coal (1,579 participants, 8.5%), and a combination of two solid fuels (193 participants, 1.0%). Based on World Health Organization (WHO) guidelines, 7,334 participants (39.3%) were classified as physically inactive, 1,864 participants (10.0%) as insufficiently active, and 9,442 participants (50.7%) as physically active.

**Table 1:** Participant characteristics based on CHARLS 2018

Characteristics	Total	Female	Male	P value ^a^
Number of participants	18,640	9735	8905	
Age (years)	61.66 (10.12)	61.44 (10.25)	61.91 (9.97)	0.002
Residence (rural, %)	11,195 (60.1)	5821 (59.8)	5374 (60.3)	0.450
Education				< 0.001
Primary school and below	12,917 (69.3)	7512 (77.2)	5405 (60.7)	
Middle school	3589 (19.3)	1432 (14.7)	2157 (24.2)	
High school and above	2134 (11.4)	791 (8.1)	1343 (15.1)	
Marital status (Married or partnered, %)	15,941 (85.5)	7912 (81.3)	8029 (90.2)	< 0.001
Household expenses per capita				0.171
Low	6215 (33.3)	3306 (34.0)	2909 (32.7)	
Median	6216 (33.3)	3211 (33.0)	3005 (33.7)	
High	6209 (33.3)	3218 (33.1)	2991 (33.6)	
Smoke (yes, %)	7940 (42.6)	767 (7.9)	7173 (80.6)	< 0.001
Drink (yes, %)	8869 (47.6)	2212 (22.7)	6657 (74.8)	< 0.001
Sleep duration (< 7 h//day)	8252 (44.3)	4772 (49.0)	3480 (39.1)	< 0.001
Installation of air cleaner	491 (2.6)	254 (2.6)	237 (2.7)	0.860
Air quality index ^b^	81.65 [68.14, 101.80]	81.65 [68.14, 101.70]	81.65 [68.65, 102.12]	0.512
CVD (yes, %)	4572 (24.5)	2642 (27.1)	1930 (21.7)	< 0.001
Solid fuel usage (yes, %)	6487 (34.8)	3373 (34.6)	3114 (35.0)	0.657
Types of fuel used				0.947
Clean energy	12,153 (65.2)	6362 (65.4)	5791 (65.0)	
Coal	1579 (8.5)	826 (8.5)	753 (8.5)	
Crop residue/Wood burning	4715 (25.3)	2445 (25.1)	2270 (25.5)	
Mixed solid fuels ^c^	193 (1.0)	102 (1.0)	91 (1.0)	
WHO physical activity guideline ^d^				< 0.001
Inactive	7334 (39.3)	3798 (39.0)	3536 (39.7)	
Insufficiently active	1864 (10.0)	1104 (11.3)	760 (8.5)	
Physically active	9442 (50.7)	4833 (49.6)	4609 (51.8)	
MVPA (hours/day)	0.71 [0.00, 2.99]	0.53 [0.00, 2.99]	0.85 [0.00, 3.43]	< 0.001

Continuous covariates were expressed as mean (standard deviation). Continuous exposure variables were expressed as median (25th, 75th percentile). Categorical variables were expressed as number (percentage). ^a^ Variables between groups were compared by t test, Kruskal-Wallis test, or Chi-square test. P < 0.05 was considered statistically significant. ^b^ The air quality index is a numerical indicator used to assess air quality, based on the concentrations of multiple pollutants, including CO, PM_2.5_, SO_2_, NO_2_, O_3_, and PM_10_. ^c^ Mixed solid fuel refers to the main solid fuels used for cooking and heating, including coal and crop residue/wood. ^d^ Based on the World Health Organization Guidelines on Physical Activity and Sedentary Behaviour 2020 and the International Physical Activity Questionnaire, inactive was considered as not participating moderate-vigorous physical activity. And we use the same METs as 300 min of moderate physical activity or 150 min of vigorous physical activity as the dividing line between insufficiently active and physically active. CVD, cardiovascular disease; MVPA, moderate-vigorous physical activity; WHO, World Health Organization

### Independent associations of MVPA and indoor pollution with the prevalence of CVD

After full adjustment, each additional 0.5 h/day of MVPA was associated with lower odds of CVD (OR (95% CI) = 0.95 (0.94–0.96)), with similar associations observed among females (OR (95% CI) = 0.95 (0.94–0.97); males (OR (95% CI) = 0.95 (0.93–0.96)) (Table 2, Supplementary Table 1). Compared with inactive individuals, both the insufficiently active (OR (95% CI) = 0.82 (0.70–0.96) for females; 0.82 (0.68–0.99) for males) and the sufficiently active (OR (95% CI) = 0.69 (0.63–0.77) for females; 0.64 (0.57–0.72) for males) were associated with lower odds of CVD.

**Table 2:** Independent association between MVPA and the prevalence of CVD, CHARLS2018

		Odds ratio (95%CI)		
	Events/*N*	Model 1 ^a^	Model 2 ^b^	Model 3 ^c^
Female	2,642/9,735			
WHO recommendation ^d^				
Inactive	1,300/3,798	Reference	Reference	Reference
Insufficiently active	295/1,104	0.79 (0.68, 0.92)	0.80 (0.69, 0.94)	0.82 (0.70, 0.96)
Physically active	1,047/4,833	0.67 (0.60, 0.74)	0.67 (0.60, 0.74)	0.69 (0.63, 0.77)
Per 0.5 h/day MVPA increase		0.95 (0.94, 0.96)	0.95 (0.94, 0.96)	0.95 (0.94, 0.97)
Male	1,930/8,905			
WHO recommendation ^d^				
Inactive	995/3,536	Reference	Reference	Reference
Insufficiently active	178/760	0.82 (0.68, 0.99)	0.82 (0.68, 0.99)	0.82 (0.68, 0.99)
Physically active	757/4,609	0.63 (0.57, 0.71)	0.63 (0.56, 0.71)	0.64 (0.57, 0.72)
Per 0.5 h/day MVPA increase		0.95 (0.93, 0.96)	0.94 (0.93, 0.96)	0.95 (0.93, 0.96)

^a^ Model.1 adjusted for age, education, residence, marital status, and tertile of household expenses per capita. ^b^ Model. 2: model. 1 further adjusted for smoke, drink, and sleep duration. ^c^ Model. 3: model. 2 further adjusted for air quality index and the installation of air cleaner. ^d^ Based on the World Health Organization Physical Activity Guidelines and the International Physical Activity Questionnaire, inactive was considered as not participating MVPA. And we use the same METs as 300 min of moderate physical activity or 150 min of vigorous physical activity as the dividing line between insufficiently active and physically active. CVD, cardiovascular disease; MVPA, moderate-vigorous physical activity; CI, confidence interval; WHO, World Health Organization; METs, metabolic equivalents

Compared with clean energy usage, solid fuel users exhibited a higher odd of CVD prevalence (OR (95% CI) = 1.42 (1.28–1.57) for females; 1.34 (1.20–1.51) for males). Moreover, we examined the differences between the use of solid fuels and clean energy sources, and found that the highest prevalence of CVD was observed among individuals using two major types of solid fuels for cooking and heating (OR (95% CI) = 4.33 (2.87–6.54) for females and 2.92 (1.87–4.57) for males), followed by those who used coal (OR (95% CI) = 1.50 (1.27–1.76) for females; 1.48 (1.24–1.77) for males) alone or crop residue/wood burning (OR (95% CI) = 1.31 (1.16–1.47) for females; 1.23 (1.08–1.41) for males) alone (Table 3, Supplementary Table 2).

**Table 3:** Independent association between solid fuel usage and the prevalence of CVD, CHARLS2018

		Odds ratio (95%CI)		
	Events/N	Model 1 ^a^	Model 2 ^b^	Model 3 ^c^
Female	2,642/9,735			
Fuel usage				
Clean energy	1,590/6,362	Reference	Reference	Reference
Solid fuels	1,052/3,373	1.44 (1.31, 1.60)	1.40 (1.27, 1.55)	1.42 (1.28, 1.57)
Types of fuel used				
Clean energy	1,590/6,362	Reference	Reference	Reference
Coal	294/826	1.65 (1.41, 1.93)	1.61 (1.37, 1.89)	1.50 (1.27, 1.76)
Crop residue/Wood burning	702/2,445	1.29 (1.15, 1.44)	1.25 (1.11, 1.40)	1.31 (1.16, 1.47)
Mixed solid fuels ^d^	56/102	4.51 (3.01, 6.76)	4.09 (2.71, 6.16)	4.33 (2.87, 6.54)
Male	1,930/8,905			
Fuel usage				
Clean energy	1,179/5,791	Reference	Reference	Reference
Solid fuels	751/3,114	1.33 (1.19, 1.49)	1.33 (1.18, 1.49)	1.34 (1.20, 1.51)
Types of fuel used				
Clean energy	1,179/5,791	Reference	Reference	Reference
Coal	221/753	1.63 (1.37, 1.93)	1.63 (1.37, 1.94)	1.48 (1.24, 1.77)
Crop residue/Wood burning	497/2,270	1.17 (1.03, 1.34)	1.16 (1.02, 1.33)	1.23 (1.08, 1.41)
Mixed solid fuels ^d^	33/91	2.63 (1.68, 4.10)	2.65 (1.70, 4.14)	2.92 (1.87, 4.57)

^a^ Model.1 adjusted for age, education, residence, marital status, and tertile of household expenses per capita. ^b^ Model. 2: model. 1 further adjusted for smoke, drink, and sleep duration. ^c^ Model. 3: model. 2 further adjusted for air quality index and the installation of air cleaner. ^d^ Mixed solid fuel refers to the main solid fuels used for cooking and heating, including coal and crop residue/wood. CVD, cardiovascular disease; MVPA, moderate-vigorous physical activity; CI, confidence interval

### Combined associations of MVPA and indoor pollution with the prevalence of CVD

Joint analysis (Table 4, Supplementary Table 3) revealed that, relative to physically active participants using clean energy, inactive individuals with solid fuel use had higher CVD prevalence. Notably, estimates for mixed fuel users were statistically unstable with wide confidence intervals, warranting confirmation in larger samples.

**Table 4:** Joint association of MVPA and solid fuel usage with the prevalence of CVD, CHARLS 2018

	Odds ratio (95%CI)			
	Clean energy	Coal	Crop residue/Wood burning	Mixed solid fuels ^b c^
Female				
Physically active ^a^	Reference	1.82 (1.41, 2.34)	1.32 (1.12, 1.56)	3.78 (2.00, 7.17)
Insufficiently active ^a^	1.16 (0.96, 1.41)	1.61 (1.04, 2.50)	1.97 (1.41, 2.76)	4.15 (1.49, 11.51)
Inactive ^a^	1.50 (1.32, 1.70)	1.92 (1.51, 2.43)	1.93 (1.62, 2.30)	7.57 (3.96, 14.48)
Male				
Physically active ^a^	Reference	1.48 (1.11, 1.97)	1.36 (1.13, 1.64)	2.70 (1.43, 5.10)
Insufficiently active ^a^	1.29 (1.02, 1.62)	1.92 (1.12, 3.29)	1.80 (1.19, 2.73)	7.83 (1.05, 58.33)
Inactive ^a^	1.64 (1.42, 1.89)	2.32 (1.81, 2.98)	1.87 (1.54, 2.28)	4.96 (2.49, 9.86)

All model adjusted for age, education, residence, marital status, tertile of household expenses per capita, smoke, drink, sleep duration, air quality index and the installation of air cleaner. ^a^ Based on the World Health Organization Physical Activity Guidelines and the International Physical Activity Questionnaire, inactive was considered as not participating MVPA. And we use the same METs as 300 min of moderate physical activity or 150 min of vigorous physical activity as the dividing line between insufficiently active and physically active. ^b^ Mixed solid fuel refers to the main solid fuels used for cooking and heating, including coal and crop residue/wood. ^c^ Wide confidence intervals were observed for the mixed solid fuel group due to its small sample size, underscoring the uncertainty of this finding. CVD, cardiovascular diseases; MVPA, moderate-vigorous physical activity; CI, confidence interval

**Fig. 1:**
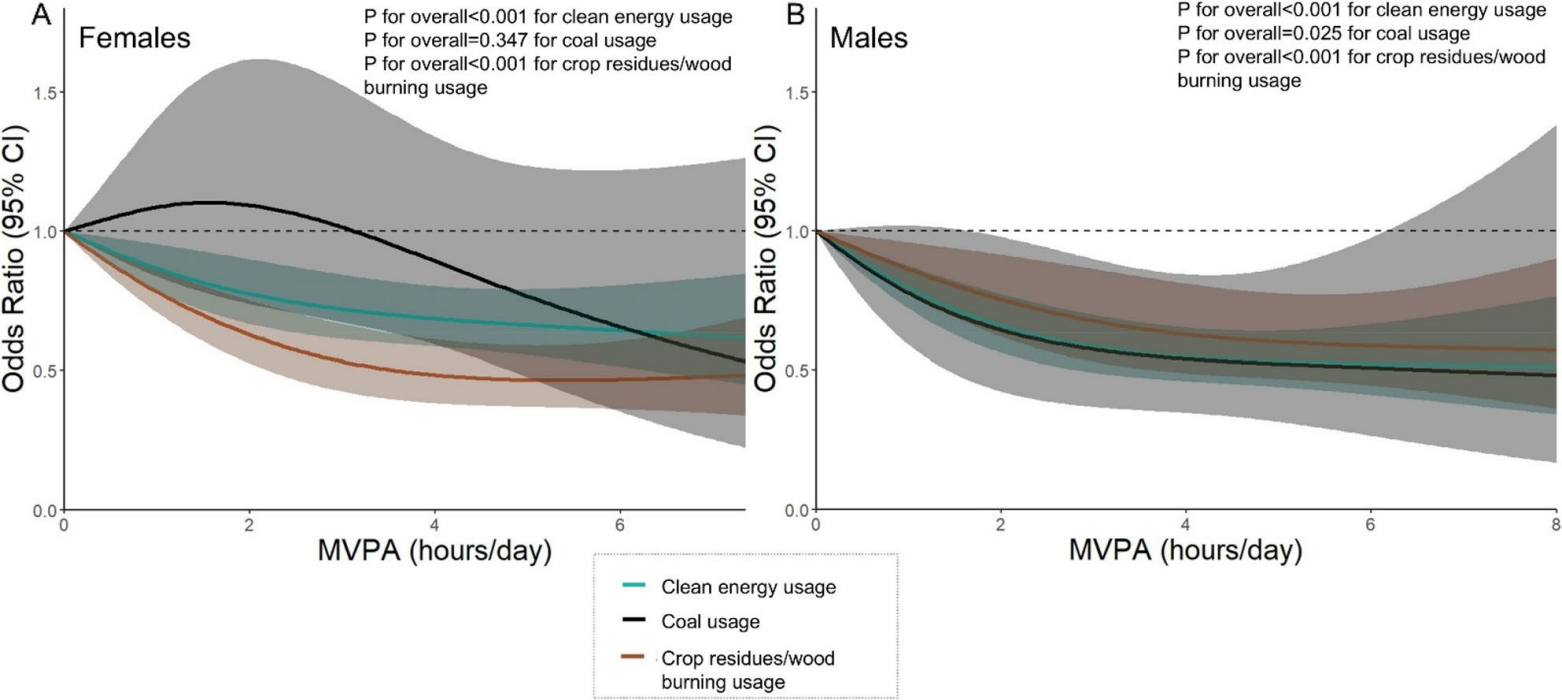
Dose–response curves of the associations between MVPA and the prevalence of CVD stratified by the types of solid fuel, CHARLS 2018. The panel depicts adjusted restricted cubic splines with 95% confidence. All models were adjusted for age, education, residence, marital status, tertile of household expenses per capita, smoke, drink, sleep duration, air quality index and the installation of air cleaner. Overall P: male clean 7.91e-11, coal 0.025, crop/wood 4.15e-4; female clean 1.16e-5, coal 0.347, crop/wood 1.18e-9. Nonlinear P: male clean 0.018, coal 0.345, crop/wood 0.232; female clean 0.176, coal 0.288, crop/wood 0.010. CVD, cardiovascular diseases; MVPA, moderate-vigorous physical activity

Dose–response analyses stratified by type of fuel use were also conducted (Fig. 1, Supplementary Table 4). We found that the protective effect of MVPA on CVD prevalence was consistent among individuals using clean energy and those using crop residue/wood burning (P for overall < 0.001). However, among individuals using coal, the inverse association of MVPA on CVD prevalence was only observed in males (P for overall < 0.05). Additionally, non-linear associations between MVPA and CVD prevalence were observed among female users of crop residue or wood, as well as among male users of clean energy (*P* < 0.05). Due to the insufficient sample size among mixed solid fuel users, dose–response modeling was not performed for this group.

### Mediation effects of MVPA on the associations between indoor pollution and the prevalence of CVD

**Fig. 2:**
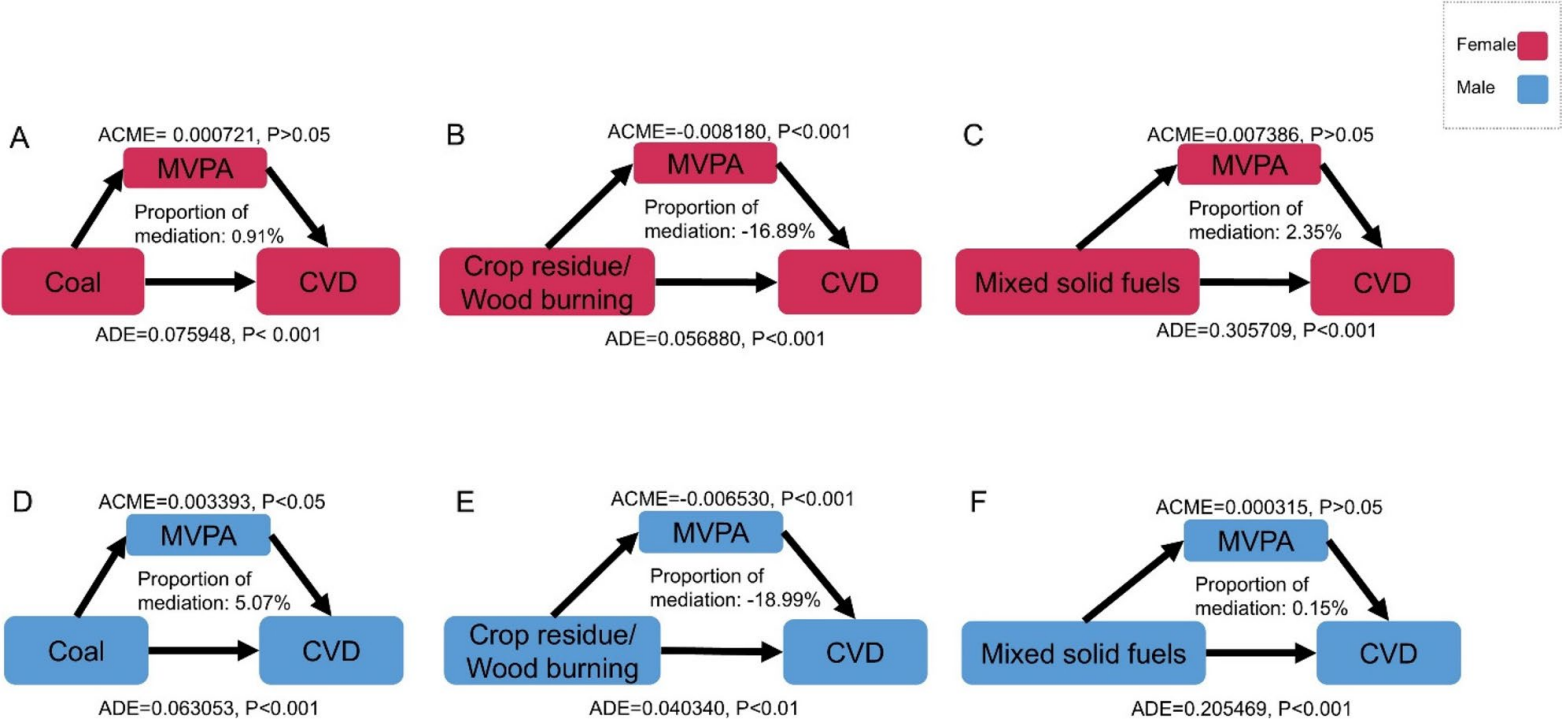
Path diagram of the data example in which MVPA is hypothesized as a mediator of the relation between the types of solid fuel and the prevalence of CVD, CHARLS 2018. All Models were adjusted for age, education, residence, marital status, tertile of household expenses per capita, smoke, drink, sleep duration, air quality index and the installation of air cleaner. CVD, cardiovascular diseases; MVPA, moderate-vigorous physical activity

MVPA exhibited significant mediating effects in the association between crop residue/wood burning and CVD prevalence in both females and males (*P* < 0.001) (Fig. 2, Supplementary Table 5). Additionally, a significant mediating effect of MVPA was also observed between coal use and CVD prevalence among males (*P* < 0.05). Compared with clean energy users, coal use was significantly associated with reduced MVPA duration among males (*P* < 0.05), while crop residue/wood burning was linked to increased MVPA duration in both sexes (*P* < 0.001) (Supplementary Fig. 2–4). In females, MVPA mediated 0.91%, − 16.89%, and 2.35% of the associations between CVD prevalence and exposure to coal, crop residue/wood burning, and mixed solid fuels, respectively. In males, the corresponding statistical mediation proportions were 5.07%, − 18.99%, and 0.15%.

## Discussion

Based on data from the nationally representative CHARLS, our study suggests that PA may modify the association between indoor pollution and CVD prevalence, with notable differences across fuel types and sex. Among females, PA was associated with a lower CVD prevalence in households using clean fuels and crop residue/wood burning; among males, this inverse statistical association was consistent across all fuel types. Furthermore, the statistical mediation proportion of PA in the association between solid fuel use and CVD prevalence differed in direction between coal and crop/wood users. This highlights the need for targeted public health interventions to protect the middle-aged and elderly amid the ongoing energy transition.

### Comparison with other studies

The burning of solid fuels (e.g., coal, wood) releases complex mixtures [40–43], which are a risk factor for CVD [44]. Previous surveys of elderly individuals and premenopausal women in developing countries have found a correlation between solid fuel use and increased blood pressure, as well as a higher prevalence of hypertension [5, 45, 46]. Moreover, solid fuel use and poor kitchen ventilation were associated with increased cardiometabolic risk among Chinese women and the elderly population [5, 47], but such risks were not observed in Peru [48]. However, in a multicenter study, solid fuel use was found to be associated with lower rates of blood pressure and hypertension compared to clean energy use [49]. These disparate results may be due to differences in the surveyed countries, which may be at different stages of development, have varying levels of economic and social conditions, and have different preferences for solid fuel use. To ensure robust estimation of associations, our analysis of individual and combined solid fuel effects incorporated adjustments for sociodemographic profiles, behavioral patterns, and ambient air pollution, using nationally representative data. Consistent with previous studies [6], our findings indicated that individuals who primarily use solid fuels for cooking or heating had a higher CVD prevalence.

Although PA is widely recognized as a beneficial strategy for reducing the risk of CVD [10–13], its role under conditions of indoor air pollution remains insufficiently investigated. Evidence from the Henan Rural Cohort Study indicates that individuals using solid fuels, compared with those using clean energy sources, exhibit poorer health outcomes [50]. However, maintaining adequate levels of PA appears to mitigate the adverse health effects associated with solid fuel use [50]. In further analyses examining specific types of solid fuels, PA remained generally associated with a lower prevalence of CVD across all fuel types examined. However, the limited sample size of participants using mixed solid fuels prevented firm conclusions for this subgroup, emphasizing the importance of dedicating future research to this vulnerable group.

In this study, dose–response analyses were conducted to examine both linear and non-linear associations between PA and CVD prevalence across different types of solid fuel use. While a significant linear inverse association between MVPA and CVD prevalence was consistently observed across most subgroups, non-linear relationships were specifically identified among female users of crop residue or wood and male users of clean energy. In many Chinese families, women relying on biomass fuels are primarily responsible for cooking [51], meaning that higher PA levels may be intrinsically linked to prolonged exposure to smoke from solid fuel combustion. This co-exposure may attenuate the benefits of PA, leading to a curvilinear relationship. Moreover, the non-linear relationship observed in males may reflect a diminishing marginal return at high PA levels—a phenomenon well documented in PA epidemiology [52]. In the mediation analysis, the negative statistical mediation effect observed among crop residue/wood users suggested that their higher levels of domestic or occupational PA may partially counteract the cardiovascular harm from air pollution. This higher PA level is likely attributable to the physically demanding tasks inherent in biomass fuel use, such as fuel collection and processing. This finding was consistent with the “PA transition” hypothesis, whereby the shift toward cleaner energy sources is paralleled by a decline in occupation-related PA [53].

### Gender differences

Gender disparities in PA participation are well-documented, with females consistently exhibiting significantly lower engagement levels than males throughout the life course [21, 54–56]. In addition, personality traits are associated with vigorous PA in men, whereas in women, personality is more predictive of moderate PA. This disparity is closely tied to traditional gender roles in the household division of labor. In conventional Chinese families, females typically shoulder greater domestic management responsibilities [51]. Such an unequal distribution of roles not only limits their opportunities to engage in other physical activities, but also increases their exposure to harmful substances emitted from solid fuel combustion [57]. Moreover, males’ limited involvement in cooking and heating activities [51]—which commonly involve the use of solid fuels—substantially reduces their exposure to these harmful emissions.

Mechanistically, the renin-angiotensin system (RAAS) exhibits significant sexual dimorphism in its functional balance, which is modulated by estrogen. Males demonstrate a predominant vasoconstrictor arm (ANG II/AT1R), attributed to higher renin activity and greater sensitivity to sodium load. In contrast, premenopausal women present a more active vasodilatory arm (ANG 1–7/AT2R) [58]. This protective pattern shifts toward vasoconstrictor dominance following menopause due to estrogen decline, thereby increasing susceptibility to hypertension in women [59, 60]. The loss of estrogen in postmenopausal women results in H₂O₂ accumulation and impaired endothelial NO synthase function, leading to endothelial dysfunction and increased cardiovascular risk [61]. Given that oxidative stress constitutes a key mechanism linking air pollution to cardiovascular disease [62], the hormone-mediated sex differences bear important pathophysiological implications. Although premenopausal women generally exhibit lower oxidative stress levels than age-matched men [63, 64], this pattern reverses after menopause, with levels surpassing those in males [65]. Furthermore, women demonstrate a blunted upregulation of PA-induced antioxidant enzymes (e.g., superoxide dismutase) compared to men [66]—enzymes critical for maintaining mitochondrial function. These sex differences may render female coal users particularly susceptible to the pro-oxidant effects of indoor air pollution, potentially offsetting the cardiovascular protection otherwise conferred by PA.

### Socio-economic disparities

While urban areas generally experience higher ambient air pollution, rural populations in developing countries face substantially greater indoor exposure due to solid fuel use [67]. This urban-rural divide reflects energy access inequalities, manifested through lower socioeconomic status, inadequate housing ventilation, and substandard cooking equipment in rural households [68]. Critically, these environmental determinants intersect with established gender norms; women’s primary responsibility for domestic tasks places them at the epicenter of this exposure [69]. Policy studies indicate that clean energy transitions could reduce indoor pollution significantly [68]. However, adoption faces challenges in rural households due to income limitations and fuel availability, resulting in the continued prevalence of “fuel stacking”—the concurrent use of clean and traditional fuels [70–72]. For female coal users, this energy poverty trap has dual implications: sustained high-level pollutant exposure alongside PA from fuel collection—a paradox that may mask the dose-response relationship between pollution and cardiovascular health. These socio-economic disparities may offer a partial explanation for our finding that the statistical association between PA and cardiovascular disease observed in men and non-coal-using women was not evident among female coal users. Their disproportionate exposure to indoor air pollution—shaped by both socioeconomic status and gendered domestic roles—might attenuate the cardiovascular benefits of PA.

The socioeconomic disparities observed in this study are not unique to China but reflect common challenges faced by many low- and middle-income countries during their energy transitions. According to the World Health Organization, approximately one-third of the global population still relies on solid fuels for cooking and heating, a problem that causes millions of premature deaths annually [4]. Similar situations have been documented in countries such as India [73], Indonesia [74], Nigeria [75]. In these regions, residents often mix clean fuels with solid fuels—a phenomenon termed “fuel stacking.” Since women typically bear primary responsibility for cooking, they consequently face greater exposure to indoor air pollution [76]. This unequal division of labor imposes additional health burdens on women. Effective interventions should therefore be multifaceted, including economic subsidies for clean energy adoption, infrastructure improvements such as kitchen ventilation upgrades, and gender-sensitive health education programs. In contrast, developed nations present a different picture. In the United States, subsidies for liquefied petroleum gas significantly accelerated the transition from solid fuels to cleaner cooking fuels [77]. The United Kingdom has implemented programs to recycle solid waste and integrate it into energy systems [78]. France is also exploring energy recovery from solid recovered fuels through gasification technology [79]. These examples demonstrate that developed nations’ response strategies emphasize technological upgrades and policy guidance to achieve comprehensive optimization of energy structures. Furthermore, interventions should also address the potential PA-energy transition paradox [53], wherein the shift to cleaner fuels may inadvertently reduce occupation-related PA. Promoting active lifestyles—such as leisure-time exercise or community-based activity programs—alongside clean energy adoption can help offset this unintended decline and maximize cardiovascular benefits.

### Strengths and limitations

To the best of our knowledge, this is the first study to examine the relationship between PA and the prevalence of CVD under conditions of indoor air pollution. Our study offers a multidimensional perspective by integrating the WHO Guidelines on PA and Sedentary Behaviour to jointly assess varying levels of PA and fuel usage, alongside exposure-response analyses related to CVD prevalence. Furthermore, mediation analyses were conducted to explore the potential role of PA in mediating the statistical association between solid fuel use and CVD prevalence.

Several limitations of this study should be acknowledged. First, due to its cross-sectional design, causal relationships cannot be established. Future longitudinal research is necessary to clarify potential bidirectional associations and temporal sequences. Second, PA, CVD diagnosis, and household fuel type were all self-reported, which may introduce recall bias, social desirability bias, and potential misclassification. Third, our analysis focused on overall PA levels without differentiating activity contexts (e.g., indoor vs. outdoor), which could influence pollutant exposure differently. Future research is warranted to employ location-aware assessment tools (e.g., accelerometry with GPS, time-activity diaries) to capture both the intensity and context of PA in relation to pollutant exposure. Fourth, given that different activity domains have distinct cardiovascular impacts [80], further studies should employ objective measurement tools and context-specific assessments to better explore these complex interactions. Finally, the CHARLS dataset lacks detailed information on fuel use patterns (e.g., frequency and duration), which may lead to exposure misclassification. Future studies incorporating more refined exposure assessment are warranted to confirm our findings.

## Conclusion

Our findings suggest that the cardiovascular benefits of PA may be influenced by the type of fuel used. Regardless of fuel type, physically active individuals exhibited lower odds of CVD prevalence compared to their inactive counterparts in both sexes. However, the inverse association between PA and CVD prevalence remained statistically significant among males across all solid fuel types, whereas among females, it was significant only for users of clean energy and crop residue/wood burning. Moreover, PA statistically mediated the association between solid fuel use and CVD prevalence, but the direction of this mediation differed between coal and crop residue/wood burning users. These findings underscore the need for targeted public health policies to protect middle-aged and elderly populations during energy transitions.

## Supplementary Information

Supplementary Material 1.

## Data Availability

The CHARLS database used in this study can be accessed by researchers on application.

## Abbreviations

CVD: Cardiovascular disease
PA: Physical activity
MVPA: Moderate-vigorous physical activity
METs: Metabolic equivalents
WHO: World Health Organization
PM: Fine particulate matter
CO: Carbon monoxide
AQI: Air quality index
ORs: Odds ratios
CIs: Confidence intervals
ROS: reactive oxygen species
MPO: Myeloperoxidase
HDL: High-density lipoprotein
