# Supplementary material for: The association between physical activity and cardiovascular disease under indoor air pollution among middle-aged-to-elderly people: a sex-specific analysis from CHARLS
Source: BMC Public Health. 2026 Mar 30;26:1513. doi: 10.1186/s12889-026-27175-w (PMC13159321; doi:10.1186/s12889-026-27175-w)
Supplement: Supplementary file 1 — Supplementary Material 1. [file 12889_2026_27175_MOESM1_ESM.docx]

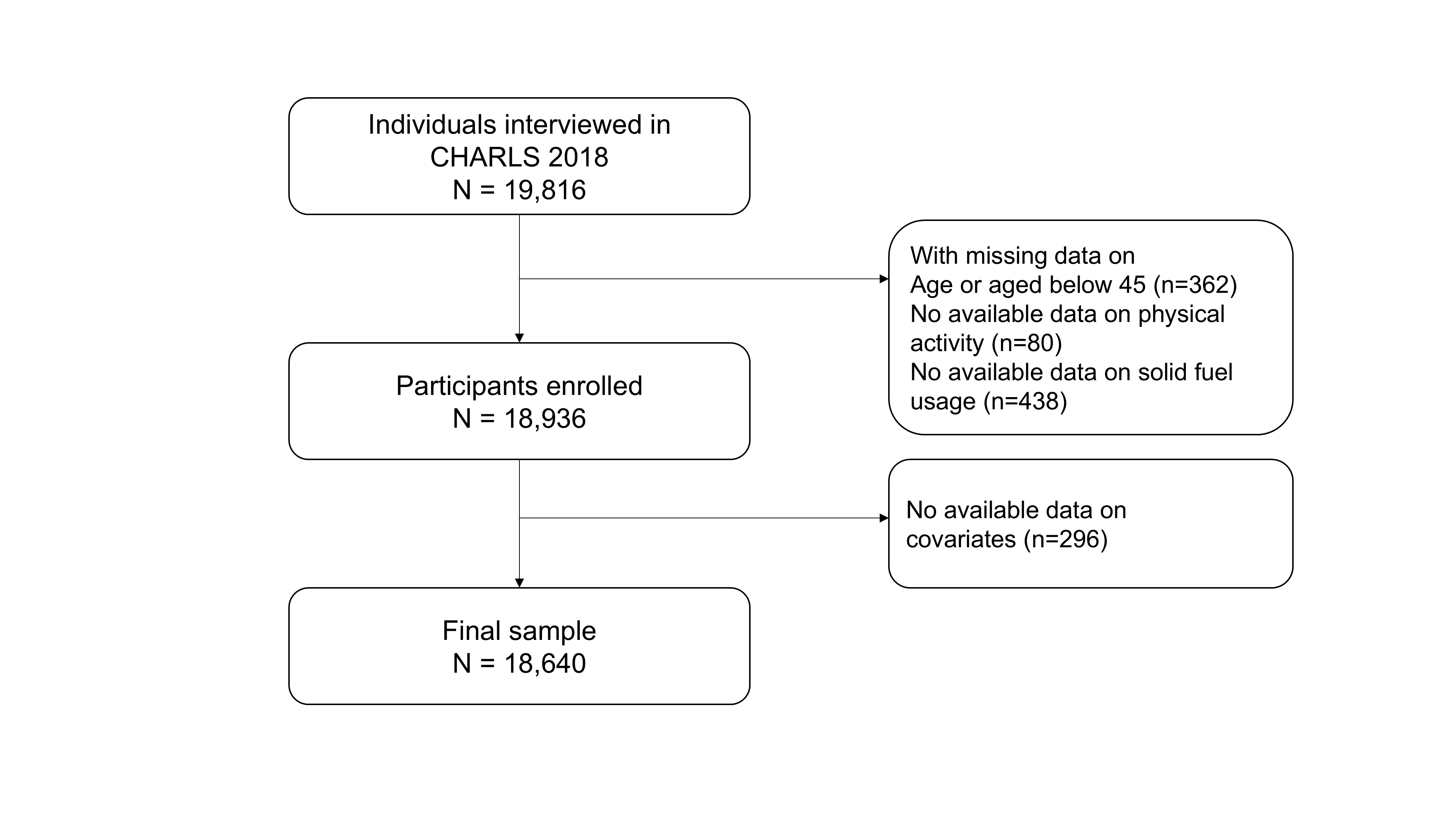


Supplementary figure 1 Flowchart of participant selection process


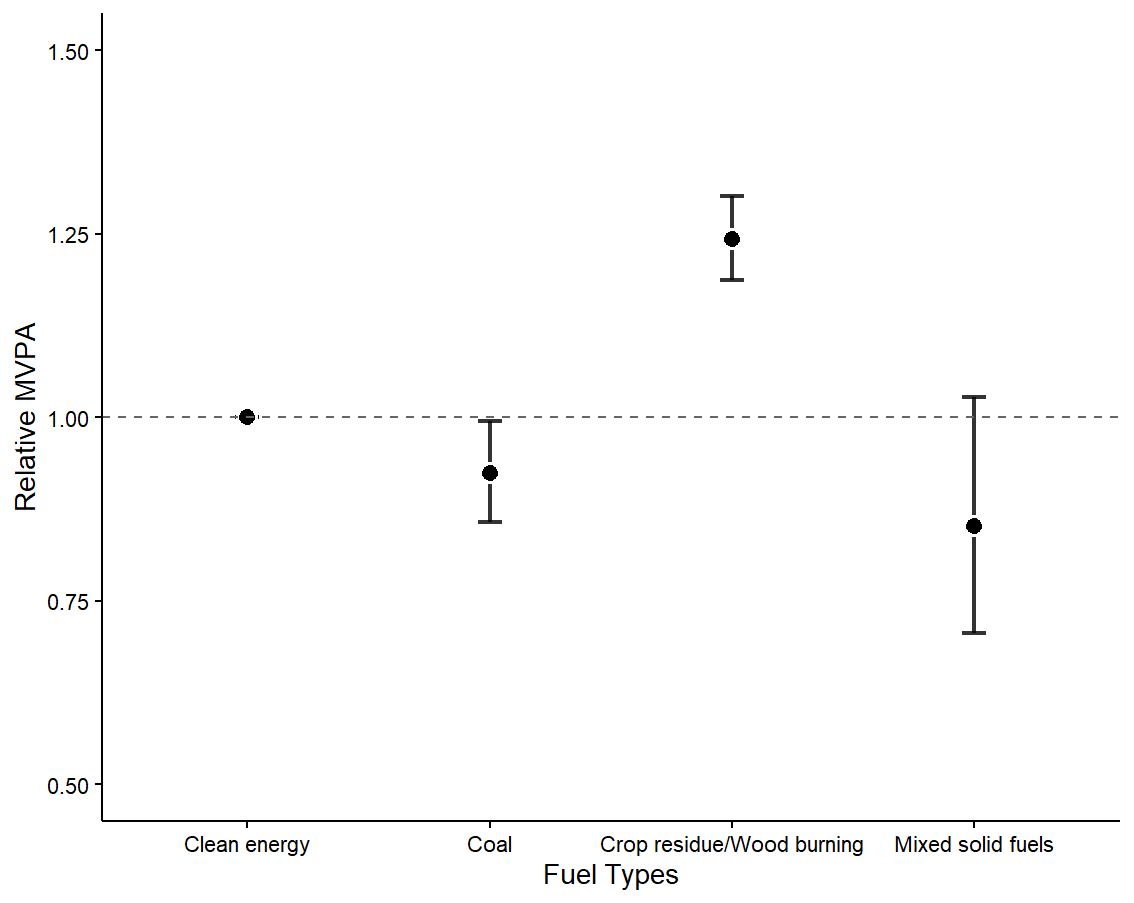


Supplementary figure 2 Independent association between the types of fuel and MVPA, CHARLS 2018. All model adjusted for sex, age, education, residence, marital status, tertile of household expenses per capita, smoke, drink, sleep duration, air quality index and the installation of air cleaner. Mixed solid fuels refer to the main solid fuels used for cooking and heating, including coal and crop residue/wood. The results present by relative MVPA and 95% confidence interval.


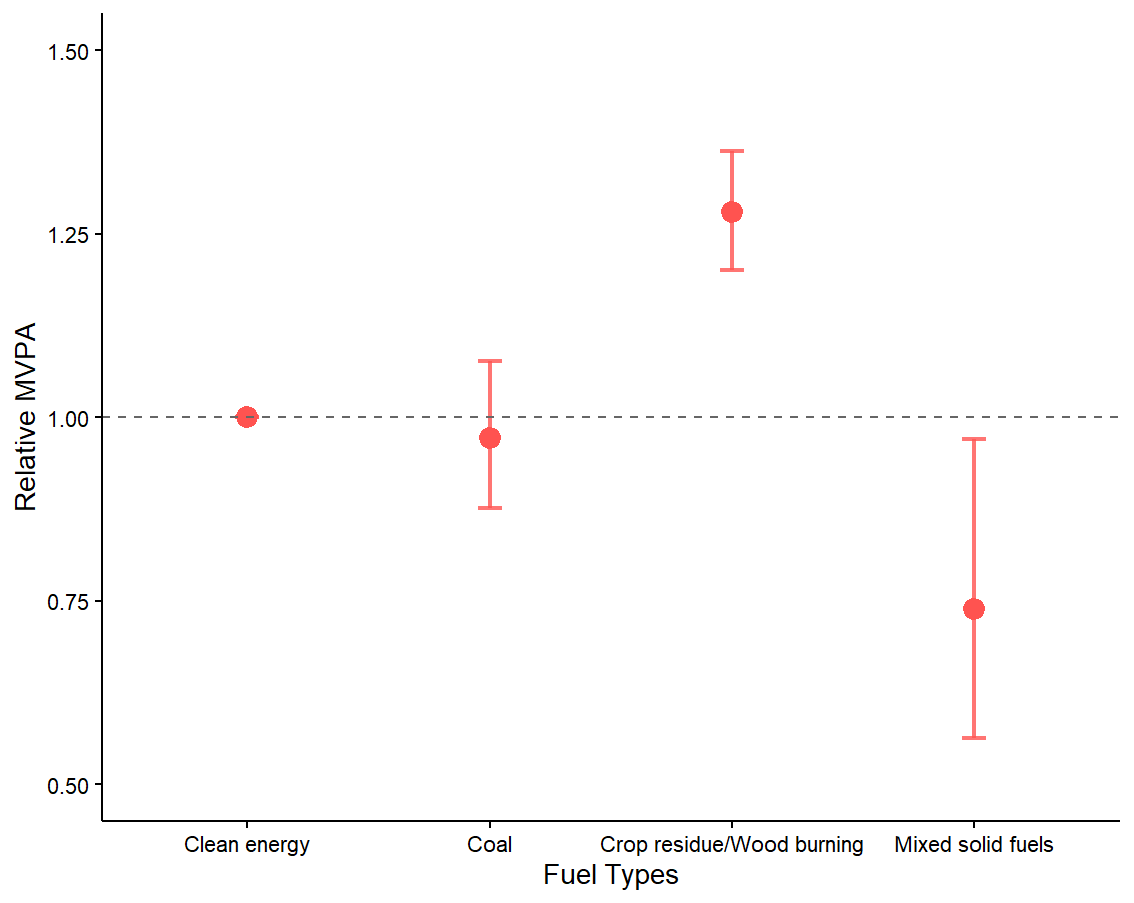


Supplementary figure 3 Independent association between the types of fuel and MVPA in females, CHARLS 2018. All model adjusted for age, education, residence, marital status, tertile of household expenses per capita, smoke, drink, sleep duration, air quality index and the installation of air cleaner. Mixed solid fuels refer to the main solid fuels used for cooking and heating, including coal and crop residue/wood. The results present by relative MVPA and 95% confidence interval.


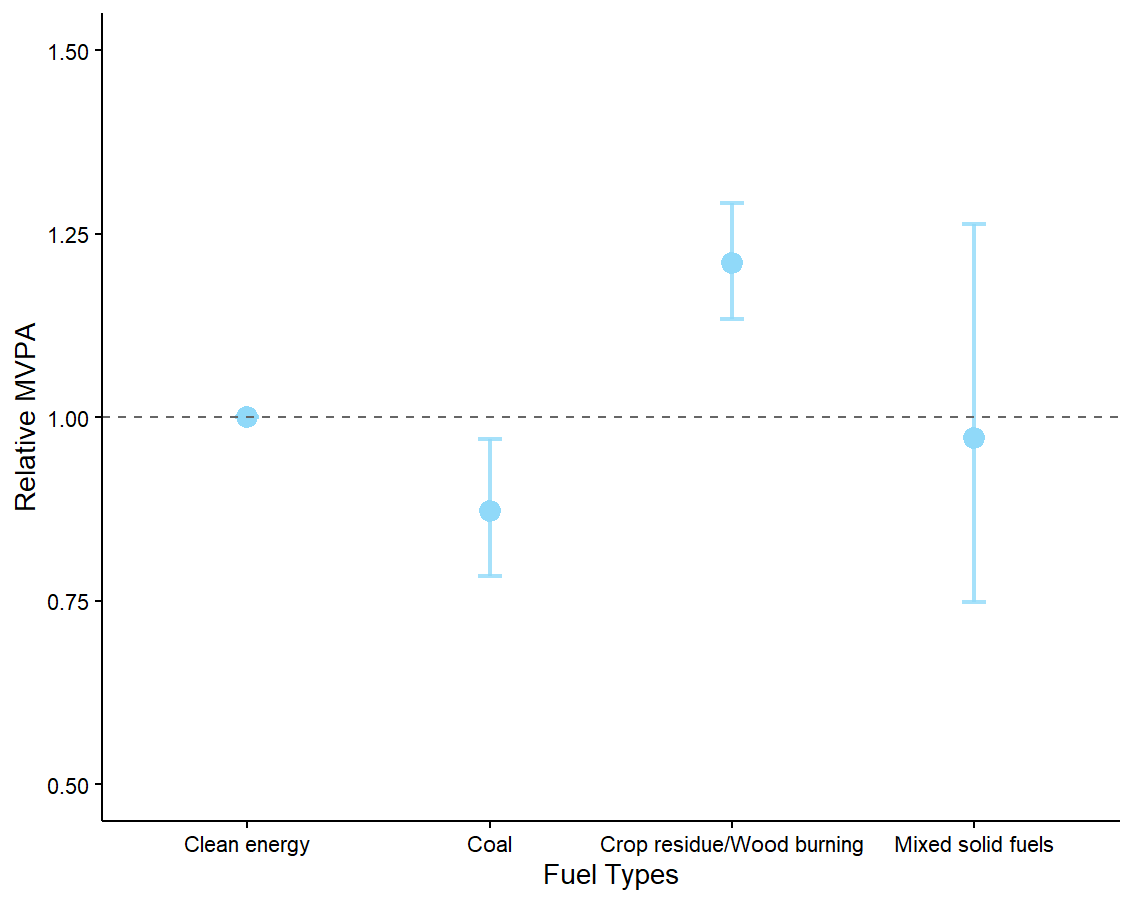


Supplementary figure 4 Independent association between the types of fuel and MVPA in males, CHARLS 2018. All model adjusted for age, education, residence, marital status, tertile of household expenses per capita, smoke, drink, sleep duration, air quality index and the installation of air cleaner. Mixed solid fuels refer to the main solid fuels used for cooking and heating, including coal and crop residue/wood. The results present by relative MVPA and 95% confidence interval.


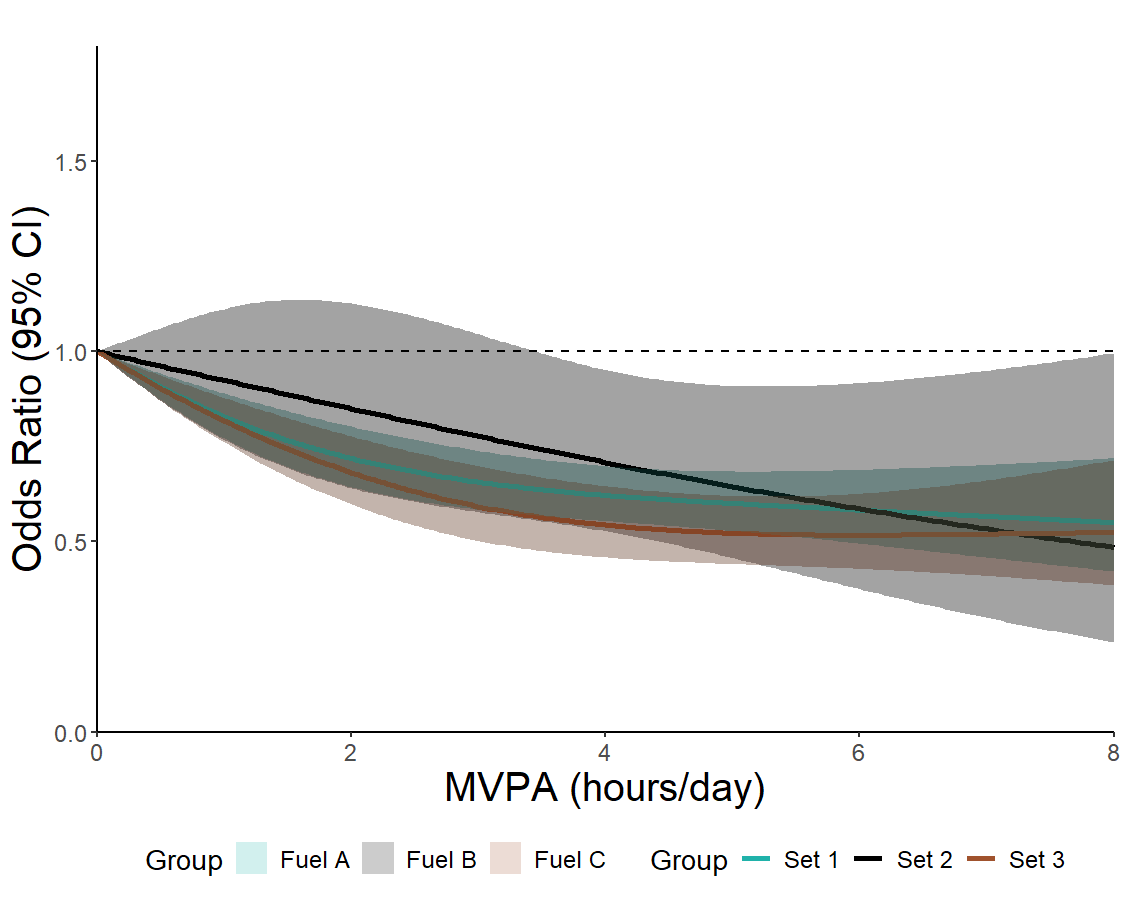


Supplementary figure 5 Dose–response curves of the associations between MVPA and the prevalence of CVD stratified by the types of solid fuel, CHARLS 2018. The panel depicts adjusted restricted cubic splines with 95% confidence. All models were adjusted for sex, age, education, residence, marital status, tertile of household expenses per capita, smoke, drink, sleep duration, air quality index and the installation of air cleaner. CVD, cardiovascular diseases; MVPA, moderate-vigorous physical activity.

| Supplementary table 1  Independent association between MVPA and the prevalence of CVD, CHARLS 2018. | | | | |
| --- | --- | --- | --- | --- |
|  |  | OR (95%CI) | | |
|  | Events/N | Model 1 ^a^ | Model 2 ^b^ | Model 3 ^c^ |
| Total | 4,572/18,640 |  |  |  |
| WHO recommendation ^d^ |  |  |  |  |
| Inactive | 2,295/7,334 | Reference | Reference | Reference |
| Insufficiently active | 473/1,864 | 0.80 (0.71, 0.90) | 0.81 (0.72, 0.91) | 0.82 (0.73, 0.92) |
| Physically active | 1,804/9,442 | 0.65 (0.60, 0.70) | 0.65 (0.60, 0.70) | 0.67 (0.62, 0.72) |
| Per 0.5h increase |  | 0.95 (0.94, 0.96) | 0.95 (0.94, 0.96) | 0.95 (0.94, 0.96) |
| ^a^ Model.1 adjusted for sex, age, education, residence, marital status, and tertile of household expenses per capita. ^b^ Model. 2: model. 1 further adjusted for smoke, drink, and sleep duration. ^c^ Model. 3: model. 2 further adjusted for air quality index and the installation of air cleaner. ^d^ Based on the World Health Organization Physical Activity Guidelines and the International Physical Activity Questionnaire, inactive was considered as not participating MVPA. And we use the same METs as 300 minutes of moderate physical activity or 150 minutes of vigorous physical activity as the dividing line between insufficiently active and physically active. CVD, cardiovascular disease; MVPA, moderate-vigorous physical activity; CI, confidence interval; WHO, World Health Organization; METs, metabolic equivalents. | | | | |

| Supplementary table 2  Independent association between solid fuel usage and the prevalence of CVD, CHARLS 2018. | | | | |
| --- | --- | --- | --- | --- |
|  |  | OR (95%CI) | | |
|  | Events/N | Model 1^a^ | Model 2^b^ | Model 3^c^ |
| Total | 4,572/18,640 |  |  |  |
| Solid fuel usage |  |  |  |  |
| no | 2,769/12,153 | Reference | Reference | Reference |
| yes | 1,803/6,487 | 1.39 (1.29, 1.50) | 1.37 (1.28, 1.48) | 1.39 (1.29, 1.50) |
| Types of fuel used |  |  |  |  |
| Clean energy | 2,769/12,153 | Reference | Reference | Reference |
| Coal | 515/1,579 | 1.64 (1.46, 1.84) | 1.63 (1.45, 1.84) | 1.50 (1.33, 1.69) |
| Crop residue/Wood burning | 1,199/4,715 | 1.23 (1.13, 1.35) | 1.21 (1.11, 1.33) | 1.28 (1.17, 1.40) |
| Mix | 89/193 | 3.52 (2.62, 4.72) | 3.44 (2.55, 4.62) | 3.70 (2.75, 4.99) |
| ^a^ Model.1 adjusted for sex, age, education, residence, marital status, and tertile of household expenses per capita. ^b^ Model. 2: model. 1 further adjusted for smoke, drink, and sleep duration. ^c^ Model. 3: model. 2 further adjusted for air quality index and the installation of air cleaner. ^d^ Mixed solid fuels refer to the main solid fuels used for cooking and heating, including coal and crop residue/wood. CVD, cardiovascular disease; MVPA, moderate-vigorous physical activity; CI, confidence interval. | | | | |

| Supplementary table 3  Joint association of MVPA and solid fuel usage with the prevalence of CVD, CHARLS 2018. | | | | |
| --- | --- | --- | --- | --- |
|  | OR (95%CI) | | | |
|  | Clean energy | Coal | Crop residue/Wood burning | Mixed solid fuels ^b^ |
| WHO recommendation^&^ |  |  |  |  |
| Physically active | Reference | 1.66 (1.38, 2.00) | 1.34 (1.19, 1.52) | 3.22 (2.07, 5.02) |
| Insufficiently active | 1.21 (1.04, 1.40) | 1.74 (1.24, 2.45) | 1.93 (1.49, 2.50) | 5.03 (2.03, 12.50) |
| Inactive | 1.56 (1.42, 1.72) | 2.12 (1.78, 2.51) | 1.91 (1.68, 2.18) | 6.40 (4.03, 10.19) |
| All model adjusted for sex, age, education, residence, marital status, tertile of household expenses per capita, smoke, drink, sleep duration, air quality index and the installation of air cleaner. ^a^ Based on the World Health Organization Physical Activity Guidelines and the International Physical Activity Questionnaire, inactive was considered as not participating MVPA. And we use the same METs as 300 minutes of moderate physical activity or 150 minutes of vigorous physical activity as the dividing line between insufficiently active and physically active. ^b^ Mixed solid fuels refer to the main solid fuels used for cooking and heating, including coal and crop residue/wood. CVD, cardiovascular diseases; MVPA, moderate-vigorous physical activity; CI, confidence interval. | | | | |

| Supplementary table 4  Dose–response associations of MVPA with the prevalence of CVD stratified by the types of solid fuel, CHARLS 2018. | | |
| --- | --- | --- |
| Types of fuel | P for overall | P for nonlinear |
| Total |  |  |
| Clean energy | <0.001 | 0.011 |
| Coal | 0.043 | 0.925 |
| Crop residue/Wood burning | <0.001 | 0.006 |
| Female |  |  |
| Clean energy | <0.001 | 0.176 |
| Coal | 0.347 | 0.288 |
| Crop residue/Wood burning | <0.001 | 0.010 |
| Male |  |  |
| Clean energy | <0.001 | 0.018 |
| Coal | 0.025 | 0.345 |
| Crop residue/Wood burning | <0.001 | 0.232 |
| All model adjusted for sex (only for total population), age, education, residence, marital status, tertile of household expenses per capita, smoke, drink, sleep duration, air quality index and the installation of air cleaner. ^a^ Mixed solid fuels refer to the main solid fuels used for cooking and heating, including coal and crop residue/wood. CVD, cardiovascular diseases; MVPA, moderate-vigorous physical activity; CI, confidence interval. | | |

| Supplementary table 5  Path data of the example in which MVPA is hypothesized as a mediator of the relation between solid fuel usage and the prevalence of CVD, CHARLS 2018. | | | | | | | | |
| --- | --- | --- | --- | --- | --- | --- | --- | --- |
| Type | | | ADE | ACME | | Proportion of mediation | | |
| Total | | |  |  | |  | | |
| Coal | | | 0.070708*** | 0.002057* | | 2.84%* | | |
| Crop residue/Wood burning | | | 0.04933*** | -0.007350*** | | -17.43*** | | |
| Mixed solid fuels ^a^ | | | 0.267238*** | 0.004369 | | 1.58% | | |
| Female | | |  |  | |  | | |
| Coal | | | 0.075948*** | 0.000721 | | 0.91% | | |
| Crop residue/Wood burning | | | 0.056880*** | -0.008180*** | | -16.89%*** | | |
| Mixed solid fuels ^a^ | | | 0.305709*** | 0.007386 | | 2.35%* | | |
| Male | | |  |  | |  | | |
| Coal | | | 0.063053*** | 0.003393* | | 5.07%* | | |
| Crop residue/Wood burning | | | 0.040340** | -0.006530*** | | -18.99%** | | |
| Mixed solid fuels ^a^ | | | 0.205469*** | 0.000315 | | 0.15% | | |
| *P<0.05, **P<0.01, ***P<0.001. All model adjusted for sex (only for total population), age, education, residence, marital status, tertile of household expenses per capita, smoke, drink, sleep duration, air quality index and the installation of air cleaner. ^a^ Mixed solid fuels refer to the main solid fuels used for cooking and heating, including coal and crop residue/wood. CVD, cardiovascular diseases; MVPA, moderate-vigorous physical activity; CI, confidence interval. | | | | | | | | |
| Supplementary table 6  Independent association between MVPA and the prevalence of CVD, CHARLS 2018 (Using alternative PA cut-points). | | | | | | | |  |
|  |  | OR (95%CI) | | | | | |  |
|  | Events/N | Model 1 ^a^ | | | Model 2 ^b^ | | Model 3 ^c^ |  |
| Total | 4,572/18,640 |  | | |  | |  |  |
| WHO recommendation ^d^ |  |  | | |  | |  |  |
| Inactive | 2,295/7,334 | Reference | | | Reference | | Reference |  |
| Insufficiently active | 1315/5,623 | 0.76 (0.70, 0.82) | | | 0.76 (0.70, 0.82) | | 0.77 (0.71, 0.84) |  |
| Physically active | 962/4,721 | 0.59 (0.54, 0.65) | | | 0.59 (0.54, 0.64) | | 0.61 (0.56, 0.67) |  |
| Per 0.5h increase |  | 0.95 (0.94, 0.96) | | | 0.95 (0.94, 0.96) | | 0.95 (0.94, 0.96) |  |
| Female | 2,642/9,735 |  | | |  | |  |  |
| WHO recommendation ^d^ |  |  | | |  | |  |  |
| Inactive | 1,300/3,798 | Reference | | | Reference | | Reference |  |
| Insufficiently active | 798/3,180 | 0.75 (0.67, 0.84) | | | 0.76 (0.68, 0.85) | | 0.78 (0.70, 0.87) |  |
| Physically active | 544/2,757 | 0.62 (0.55, 0.70) | | | 0.62 (0.55, 0.70) | | 0.64 (0.57, 0.73) |  |
| Per 0.5h increase |  | 0.95 (0.94, 0.96) | | | 0.95 (0.94, 0.96) | | 0.95 (0.94, 0.97) |  |
| Male | 1,930/8,905 |  | | |  | |  |  |
| WHO recommendation ^d^ |  |  | | |  | |  |  |
| Inactive | 995/3,536 | Reference | | | Reference | | Reference |  |
| Insufficiently active | 517/2,443 | 0.77 (0.68, 0.87) | | | 0.77 (0.68, 0.87) | | 0.77 (0.68, 0.87) |  |
| Physically active | 418/2,926 | 0.56 (0.49, 0.64) | | | 0.56 (0.49, 0.64) | | 0.57 (0.50, 0.66) |  |
| Per 0.5h increase |  | 0.95 (0.93, 0.96) | | | 0.94 (0.93, 0.96) | | 0.95 (0.93, 0.96) |  |
| ^a^ Model.1 adjusted for sex (only for total population), age, education, residence, marital status, and tertile of household expenses per capita. ^b^ Model. 2: model. 1 further adjusted for smoke, drink, and sleep duration. ^c^ Model. 3: model. 2 further adjusted for air quality index and the installation of air cleaner. ^d^ Based on the World Health Organization Physical Activity Guidelines and the International Physical Activity Questionnaire, inactive was considered as not participating MVPA. And we employed an alternative PA classification: inactive (no MVPA), insufficiently active (any MVPA below the median), and physically active (any MVPA at or above the median). CVD, cardiovascular disease; MVPA, moderate-vigorous physical activity; CI, confidence interval; WHO, World Health Organization; METs, metabolic equivalents. | | | | | | | |  |

| Supplementary table 7  Joint association of MVPA and solid fuel usage with the prevalence of CVD, CHARLS 2018 (Using alternative PA cut-points). | | | | |
| --- | --- | --- | --- | --- |
|  | Odds ratio (95%CI) | | | |
|  | Clean energy | Coal | Crop residue/Wood burning | Mixed solid fuels ^b^ |
| Total |  |  |  |  |
| Physically active ^a^ | Reference | 1.54 (1.18, 2.00) | 1.32 (1.13, 1.54) | 2.25 (1.18, 4.31) |
| Insufficiently active ^a^ | 1.22 (1.08, 1.39) | 2.03 (1.63, 2.53) | 1.83 (1.55, 2.17) | 5.45 (3.24, 9.20) |
| Inactive ^a^ | 1.69 (1.50, 1.90) | 2.29 (1.91, 2.76) | 2.06 (1.78, 2.39) | 6.90 (4.32, 11.02) |
| Female |  |  |  |  |
| Physically active ^a^ | Reference | 1.67 (1.17, 2.37) | 1.17 (0.95, 1.44) | 1.68 (0.59, 4.76) |
| Insufficiently active ^a^ | 1.09 (0.93, 1.29) | 1.90 (1.41, 2.54) | 1.80 (1.45, 2.25) | 5.96 (3.01, 11.80) |
| Inactive ^a^ | 1.53 (1.30, 1.79) | 1.96 (1.52, 2.52) | 1.97 (1.62, 2.40) | 7.71 (4.01, 14.83) |
| Male |  |  |  |  |
| Physically active ^a^ | Reference | 1.38 (0.92, 2.08) | 1.52 (1.21, 1.90) | 2.88 (1.25, 6.61) |
| Insufficiently active ^a^ | 1.43 (1.18, 1.73) | 2.19 (1.56, 3.06) | 1.81 (1.39, 2.36) | 4.13 (1.72, 9.90) |
| Inactive ^a^ | 1.91 (1.60, 2.28) | 2.71 (2.07, 3.55) | 2.16 (1.74, 2.69) | 5.71 (2.85, 11.44) |
| All model adjusted for sex (only for total population), age, education, residence, marital status, tertile of household expenses per capita, smoke, drink, sleep duration, air quality index and the installation of air cleaner. ^a^ Based on the World Health Organization Physical Activity Guidelines and the International Physical Activity Questionnaire, inactive was considered as not participating MVPA. And we employed an alternative PA classification: inactive (no MVPA), insufficiently active (any MVPA below the median), and physically active (any MVPA at or above the median). ^b^ Mixed solid fuel refers to the main solid fuels used for cooking and heating, including coal and crop residue/wood. CVD, cardiovascular diseases; MVPA, moderate-vigorous physical activity; CI, confidence interval. | | | | |

| Supplementary table 8  Path data of the example in which MVPA is hypothesized as a mediator of the relation between solid fuel usage and the prevalence of CVD, CHARLS 2018 (Using structural equation modeling as an alternative mediation approach). | | | | |
| --- | --- | --- | --- | --- |
| Type | Total effect | Direct effect | Indirect effect | Proportion of mediation |
| Total |  |  |  |  |
| Coal | 0.241*** | 0.235*** | 0.006* | 2.5% |
| Crop residue/Wood burning | 0.149*** | 0.172*** | -0.024*** | -16.1% |
| Mixed solid fuels ^a^ | 0.788*** | 0.776*** | 0.012 | 1.5% |
| Female |  |  |  |  |
| Coal | 0.240*** | 0.238*** | 0.002 | 0.8% |
| Crop residue/Wood burning | 0.161*** | 0.186*** | -0.025*** | -15.5% |
| Mixed solid fuels ^a^ | 0.887*** | 0.867*** | 0.021* | 2.4% |
| Male |  |  |  |  |
| Coal | 0.233*** | 0.222*** | 0.011* | 4.7% |
| Crop residue/Wood burning | 0.128** | 0.150*** | -0.022*** | -17.2% |
| Mixed solid fuels ^a^ | 0.637*** | 0.636*** | 0.000 | 0.0% |
| *P<0.05, **P<0.01, ***P<0.001. All model adjusted for sex (only for total population), age, education, residence, marital status, tertile of household expenses per capita, smoke, drink, sleep duration, air quality index and the installation of air cleaner. ^a^ Mixed solid fuels refer to the main solid fuels used for cooking and heating, including coal and crop residue/wood. CVD, cardiovascular diseases; MVPA, moderate-vigorous physical activity; CI, confidence interval. | | | | |

| Supplementary table 9  Joint association of MVPA and solid fuel usage with the prevalence of CVD, CHARLS 2018 (Add another covariate). | | | | |
| --- | --- | --- | --- | --- |
|  | Odds ratio (95%CI) | | | |
|  | Clean energy | Coal | Crop residue/Wood burning | Mixed solid fuels ^b^ |
| Total |  |  |  |  |
| Physically active ^a^ | Reference | 1.62 (1.34, 1.95) | 1.32 (1.16, 1.49) | 3.42 (2.18, 5.34) |
| Insufficiently active ^a^ | 1.20 (1.04, 1.39) | 1.74 (1.24, 2.45) | 1.84 (1.42, 2.40) | 5.06 (2.04, 12.57) |
| Inactive ^a^ | 1.55 (1.41, 1.71) | 2.11 (1.78, 2.51) | 1.92 (1.68, 2.20) | 6.41 (4.02, 10.19) |
| Female |  |  |  |  |
| Physically active ^a^ | Reference | 1.77 (1.37, 2.29) | 1.32 (1.12, 1.56) | 4.12 (2.14, 7.92) |
| Insufficiently active ^a^ | 1.16 (0.96, 1.41) | 1.63 (1.05, 2.53) | 1.88 (1.34, 2.64) | 4.17 (1.50, 11.58) |
| Inactive ^a^ | 1.49 (1.31, 1.70) | 1.92 (1.51, 2.43) | 1.96 (1.63, 2.34) | 7.62 (3.98, 14.58) |
| Male |  |  |  |  |
| Physically active ^a^ | Reference | 1.44 (1.08, 1.92) | 1.31 (1.08, 1.58) | 2.81 (1.48, 5.33) |
| Insufficiently active ^a^ | 1.27 (1.00, 1.60) | 1.89 (1.10, 3.24) | 1.74 (1.14, 2.65) | 7.89 (1.06, 58.77) |
| Inactive ^a^ | 1.63 (1.41, 1.88) | 2.32 (1.80, 2.98) | 1.88 (1.53, 2.29) | 4.92 (2.47, 9.81) |
| All model adjusted for sex (only for total population), age, education, residence, marital status, tertile of household expenses per capita, smoke, drink, sleep duration, air quality index, the installation of air cleaner and ventilation situation. ^a^ Based on the World Health Organization Physical Activity Guidelines and the International Physical Activity Questionnaire, inactive was considered as not participating MVPA. And we use the same METs as 300 minutes of moderate physical activity or 150 minutes of vigorous physical activity as the dividing line between insufficiently active and physically active. ^b^ Mixed solid fuel refers to the main solid fuels used for cooking and heating, including coal and crop residue/wood. CVD, cardiovascular diseases; MVPA, moderate-vigorous physical activity; CI, confidence interval. | | | | |

| Supplementary table 10  Dose–response associations of MVPA with the prevalence of CVD stratified by the types of solid fuel, CHARLS 2018 (Add another covariate). | | |
| --- | --- | --- |
| Types of fuel | P for overall | P for nonlinear |
| Total |  |  |
| Clean energy | <0.001 | 0.011 |
| Coal | 0.0349 | 0.834 |
| Crop residue/Wood burning | <0.001 | 0.008 |
| Female |  |  |
| Clean energy | <0.001 | 0.161 |
| Coal | 0.3828 | 0.420 |
| Crop residue/Wood burning | <0.001 | 0.028 |
| Male |  |  |
| Clean energy | <0.001 | 0.022 |
| Coal | 0.0215 | 0.272 |
| Crop residue/Wood burning | <0.001 | 0.143 |
| All model adjusted for sex (only for total population), age, education, residence, marital status, tertile of household expenses per capita, smoke, drink, sleep duration, air quality index air quality index, the installation of air cleaner and ventilation situation. ^a^ Mixed solid fuels refer to the main solid fuels used for cooking and heating, including coal and crop residue/wood. CVD, cardiovascular diseases; MVPA, moderate-vigorous physical activity; CI, confidence interval. | | |

| Supplementary table 11  Path data of the example in which MVPA is hypothesized as a mediator of the relation between solid fuel usage and the prevalence of CVD, CHARLS 2018. (Add another covariate) | | | |
| --- | --- | --- | --- |
| Type | ADE | ACME | Proportion of mediation |
| Total |  |  |  |
| Coal | 0.068733*** | 0.002128** | 2.97%** |
| Crop residue/Wood burning | 0.048070*** | -0.007410*** | -18.21%*** |
| Mixed solid fuels ^a^ | 0.274401*** | 0.004579 | 1.61% |
| Female |  |  |  |
| Coal | 0.074585*** | 0.000782 | 1.03% |
| Crop residue/Wood burning | 0.056840*** | -0.008300*** | -17.02*** |
| Mixed solid fuels ^a^ | 0.316731*** | 0.007586* | 2.26%* |
| Male |  |  |  |
| Coal | 0.060937*** | 0.003534** | 5.49%** |
| Crop residue/Wood burning | 0.036690** | -0.006420*** | -21.18%** |
| Mixed solid fuels ^a^ | 0.208919*** | 0.000692 | 0.25% |
| *P<0.05, **P<0.01, ***P<0.001. All model adjusted for sex (only for total population), age, education, residence, marital status, tertile of household expenses per capita, smoke, drink, sleep duration, air quality index, the installation of air cleaner and ventilation situation. ^a^ Mixed solid fuels refer to the main solid fuels used for cooking and heating, including coal and crop residue/wood. CVD, cardiovascular diseases; MVPA, moderate-vigorous physical activity; CI, confidence interval. | | | |

| Supplementary table 12  Joint association of MVPA and solid fuel usage with the prevalence of CVD, CHARLS 2018 (Individual weights were further adjusted). | | | | |
| --- | --- | --- | --- | --- |
|  | Odds ratio (95%CI) | | | |
|  | Clean energy | Coal | Crop residue/Wood burning | Mixed solid fuels ^b^ |
| Total |  |  |  |  |
| Physically active ^a^ | Reference | 1.77 (1.44, 2.18) | 1.30 (1.14, 1.49) | 3.11 (1.85, 5.23) |
| Insufficiently active ^a^ | 1.16 (0.98, 1.37) | 1.67 (1.13, 2.48) | 1.74 (1.31, 2.32) | 4.88 (1.84, 12.98) |
| Inactive ^a^ | 1.56 (1.40, 1.74) | 2.05 (1.68, 2.50) | 1.79 (1.54, 2.07) | 4.76 (2.76, 8.22) |
| Female |  |  |  |  |
| Physically active ^a^ | Reference | 1.94 (1.47, 2.57) | 1.25 (1.04, 1.49) | 4.81 (2.20, 10.50) |
| Insufficiently active ^a^ | 1.13 (0.91, 1.39) | 1.49 (0.88, 2.52) | 1.77 (1.24, 2.53) | 4.54 (1.44, 14.28) |
| Inactive ^a^ | 1.53 (1.32, 1.77) | 1.84 (1.40, 2.42) | 1.78 (1.45, 2.18) | 5.38 (2.55, 11.35) |
| Male |  |  |  |  |
| Physically active ^a^ | Reference | 1.59 (1.15, 2.18) | 1.37 (1.12, 1.68) | 1.87 (0.88, 3.99) |
| Insufficiently active ^a^ | 1.21 (0.93, 1.58) | 1.93 (1.06, 3.49) | 1.65 (1.03, 2.64) | 4.98 (0.88, 28.21) |
| Inactive ^a^ | 1.61 (1.36, 1.89) | 2.27 (1.70, 3.02) | 1.80 (1.44, 2.24) | 3.97 (1.75, 9.00) |
| All model adjusted for age (only for total population), education, residence, marital status, tertile of household expenses per capita, smoke, drink, sleep duration, air quality index and the installation of air cleaner. ^a^ Based on the World Health Organization Physical Activity Guidelines and the International Physical Activity Questionnaire, inactive was considered as not participating MVPA. And we use the same METs as 300 minutes of moderate physical activity or 150 minutes of vigorous physical activity as the dividing line between insufficiently active and physically active. ^b^ Mixed solid fuel refers to the main solid fuels used for cooking and heating, including coal and crop residue/wood. CVD, cardiovascular diseases; MVPA, moderate-vigorous physical activity; CI, confidence interval. | | | | |

| Supplementary table 13  Dose–response associations of MVPA with the prevalence of CVD stratified by the types of solid fuel, CHARLS 2018 (Individual weights were further adjusted). | | | | |  |  |
| --- | --- | --- | --- | --- | --- | --- |
| Types of fuel | P for overall | | P for nonlinear | |  |  |
| Total |  | |  | |  |  |
| Clean energy | <0.001 | | <0.001 | |  |  |
| Coal | 0.045 | | 0.393 | |  |  |
| Crop residue/Wood burning | <0.001 | | 0.065 | |  |  |
| Female |  | |  | |  |  |
| Clean energy | <0.001 | | 0.003 | |  |  |
| Coal | 0.239 | | 0.121 | |  |  |
| Crop residue/Wood burning | <0.001 | | 0.182 | |  |  |
| Male |  | |  | |  |  |
| Clean energy | <0.001 | | 0.035 | |  |  |
| Coal | 0.034 | | 0.670 | |  |  |
| Crop residue/Wood burning | 0.003 | | 0.233 | |  |  |
| All model adjusted for sex (only for total population), age, education, residence, marital status, tertile of household expenses per capita, smoke, drink, sleep duration, air quality index air quality index and the installation of air cleaner. ^a^ Mixed solid fuels refer to the main solid fuels used for cooking and heating, including coal and crop residue/wood. CVD, cardiovascular diseases; MVPA, moderate-vigorous physical activity; CI, confidence interval. | | | | |  |  |
|  | | | | |  |  |
| Supplementary table 14  Path data of the example in which MVPA is hypothesized as a mediator of the relation between solid fuel usage and the prevalence of CVD, CHARLS 2018 (Individual weights were further adjusted). | | | | | | |
| Type | | ADE | | ACME | | Proportion of mediation |
| Total | |  | |  | |  |
| Coal | | 0.073149*** | | 0.002087* | | 2.68%* |
| Crop residue/Wood burning | | 0.041010*** | | -0.006940*** | | -20.05%*** |
| Mixed solid fuels ^a^ | | 0.234373*** | | 0.005954* | | 2.44%* |
| Female | |  | |  | |  |
| Coal | | 0.076000*** | | 0.000028 | | 0.00% |
| Crop residue/Wood burning | | 0.040880** | | -0.007500*** | | -22.29%** |
| Mixed solid fuels ^a^ | | 0.299480*** | | 0.008080** | | 2.59%** |
| Male | |  | |  | |  |
| Coal | | 0.068840*** | | 0.004370*** | | 5.87%*** |
| Crop residue/Wood burning | | 0.039870** | | -0.006040*** | | -17.67%** |
| Mixed solid fuels ^a^ | | 0.150570** | | 0.003560 | | 2.30% |
| *P<0.05, **P<0.01, ***P<0.001. All model adjusted for sex (only for total population), age, education, residence, marital status, tertile of household expenses per capita, smoke, drink, sleep duration, air quality index and the installation of air cleaner. ^a^ Mixed solid fuels refer to the main solid fuels used for cooking and heating, including coal and crop residue/wood. CVD, cardiovascular diseases; MVPA, moderate-vigorous physical activity; CI, confidence interval. | | | | | | |
